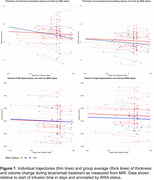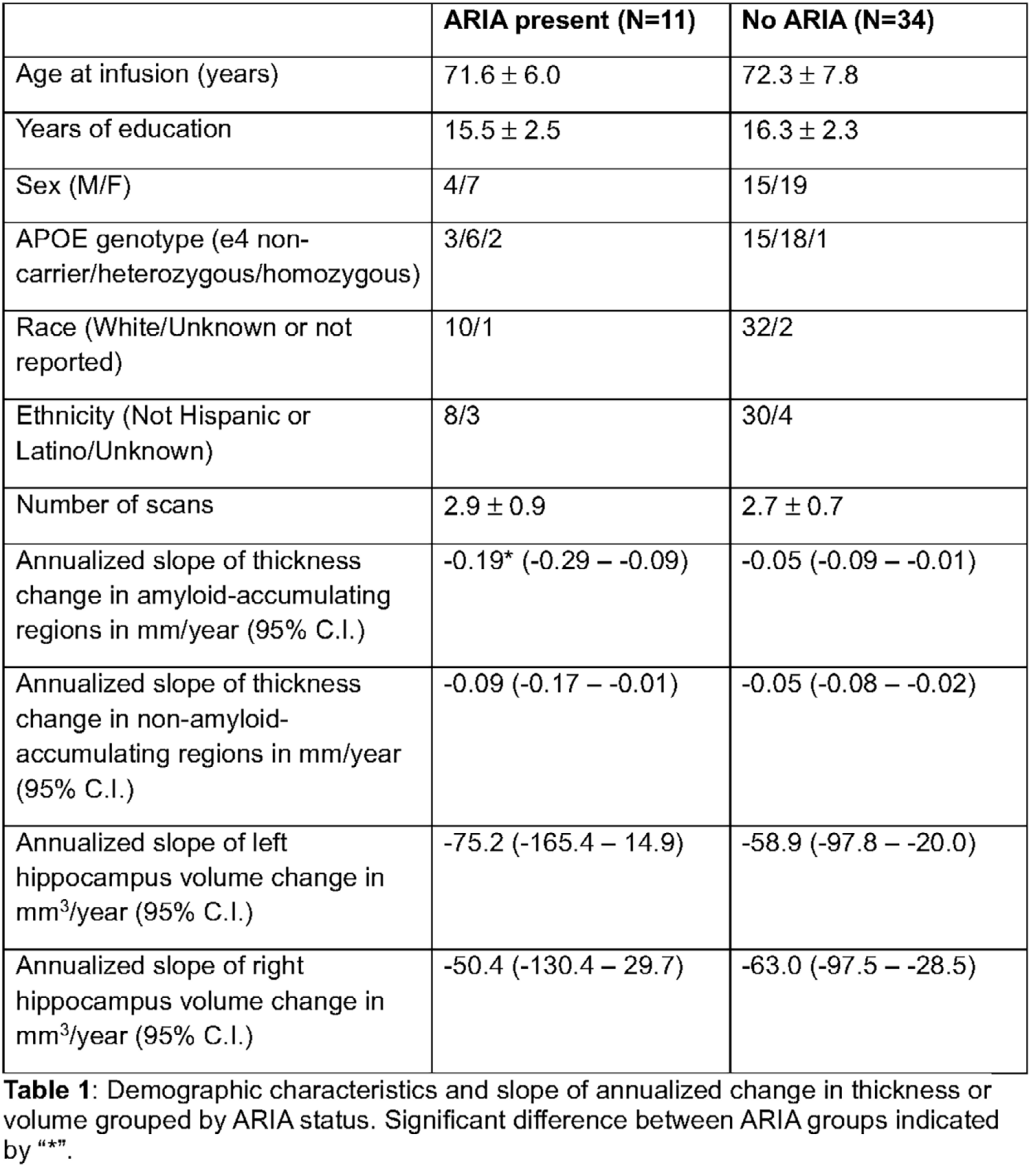# Amyloid‐accumulating cortical regions show selectively greater longitudinal thinning in the presence of ARIA during anti‐amyloid therapy

**DOI:** 10.1002/alz70861_108773

**Published:** 2025-12-23

**Authors:** Sandhitsu R Das, Christopher D. Brown, Philip A. Cook, Emily McGrew, Danielle Hing, James C. Gee, Ilya M. Nasrallah, Dawn Mechanic‐Hamilton, Paul A. Yushkevich, David A. Wolk

**Affiliations:** ^1^ University of Pennsylvania, Philadelphia, PA USA; ^2^ Penn Image Computing & Science Laboratory, Department of Radiology, University of Pennsylvania, Philadelphia, PA USA; ^3^ Perelman School of Medicine, University of Pennsylvania, Philadelphia, PA USA; ^4^ Penn Image Computing and Science Laboratory, Philadelphia, PA USA; ^5^ Department of Neurology, Perelman School of Medicine, University of Pennsylvania, Philadelphia, PA USA

## Abstract

**Background:**

Paradoxical reductions in brain volume in individuals with Alzheimer’s disease treated with anti‐amyloid therapy have been reported in clinical trials. It has been hypothesized that these changes could partly be attributed to processes related to the clearance of amyloid plaques. Further, the presence of amyloid‐related imaging abnormalities (ARIA) has been associated with greater ventricular expansion in clinical trials of anti‐amyloid immunotherapy, and may also be an important factor driving brain volume loss. Here we measure longitudinal changes from structural MRI during the course of anti‐amyloid treatment in brain regions known to accumulate amyloid plaques and ask whether these regions show differences in observed changes depending on presence of ARIA.

**Method:**

We analyzed 125 MRI images from 45 patients undergoing surveillance scanning during the course of lecanemab therapy. Average duration between the earliest and latest scans was 181 days. For each patient, images were included if from an identical MRI protocol and scanner. Measures of gray matter thickness in 100 brain regions were obtained using an unbiased longitudinal pipeline. Regions were nominally dichotomized into amyloid‐accumulating and non‐amyloid‐accumulating based on composite regions used for determining amyloid positivity from PET imaging. Thickness was analyzed using linear mixed effects model that included time and presence of ARIA (ARIA‐E or ARIA‐H) as explanatory variables and age and sex as nuisance covariates.

**Result:**

Both amyloid‐accumulating and non‐amyloid‐accumulating regions showed thinning during the treatment period. However, there was a significant interaction between time and ARIA status only in amyloid‐accumulating regions such that patients with ARIA showed greater observed thinning. Hippocampal volume also showed a decrease over time, but there was no effect of presence of ARIA.

**Conclusion:**

These data show that presence of ARIA may play a role in observed brain parenchymal changes during anti‐amyloid treatment. The specificity of the effects in amyloid‐accumulating brain regions point to an interaction between amyloid clearance and the emergence of ARIA as confounding factors in measuring longitudinal change. While the underlying mechanism remains unclear, better characterization of such effects can help disentangle their influence on MRI‐based measures of change and ultimately improve the usability of MRI as a marker for tracking potential treatment effects.